# Endoscopic ultrasound guided gastrojejunostomy in the treatment of gastric outlet obstruction: multi-centre experience from the United Kingdom

**DOI:** 10.1007/s00464-022-09692-y

**Published:** 2022-10-10

**Authors:** Wei On, Matthew T. Huggett, Alistair Young, James Pine, Andrew M. Smith, Nadeem Tehami, Ben Maher, Stephen P. Pereira, Gavin Johnson, Bharat Paranandi

**Affiliations:** 1 Department of Gastroenterology, Leeds Teaching Hospitals NHS Trust, Leeds, UK; 2 Department of Surgery, Leeds Teaching Hospitals NHS Trust, Leeds, UK; 3 Department of Gastroenterology, University Hospital Southampton NHS Foundation Trust, Southampton, UK; 4 Department of Interventional Radiology, University Hospital Southampton NHS Foundation Trust, Southampton, UK; 5 Department of Gastroenterology, University College Hospitals NHS Foundation Trust, London, UK; 6 St James’s University Hospital, 4th Floor, Bexley Wing, Beckett Street, Leeds, LS9 7TF UK

**Keywords:** Endoscopic ultrasound, Interventional endoscopy, Gastrojejunostomy, Gastric outlet obstruction

## Abstract

**Background:**

Endoscopic ultrasound guided gastrojejunostomy (EUS-GJ) with lumen apposing metal stents has recently emerged as a viable option, as an alternative to surgical gastrojejunostomy and endoscopic enteral stenting, for managing gastric outlet obstruction (GOO). We aim to perform a retrospective analysis of the efficacy, safety and outcomes of EUS-GJ performed at three tertiary institutions in the United Kingdom.

**Methods:**

Consecutive patients who underwent EUS-GJ between August 2018 and March 2021 were identified from a prospectively maintained database. Data were obtained from interrogation of electronic health records.

**Results:**

Twenty five patients (15 males) with a median age of 63 years old (range 29–80) were included for analysis. 88% (22/25) of patients had GOO due to underlying malignant disease. All patients were deemed surgically inoperable or at high surgical risk. Both technical and clinical success were achieved in 92% (23/25) of patients. There was an improvement in the mean Gastric Outlet Obstruction Scoring System scores following a technically successful EUS-GJ (2.52 vs 0.68, *p* < 0.01). Adverse events occurred in 2/25 patients (8%), both due to stent maldeployment necessitating endoscopic closure of the gastric defect with clips. Long-term follow-up data were available for 21 of 23 patients and the re-intervention rate was 4.8% (1/21) over a median follow-up period of 162 (range 5–474) days.

**Conclusion:**

EUS-GJ in carefully selected patients is an effective and safe procedure when performed by experienced endoscopists.

**Supplementary Information:**

The online version contains supplementary material available at 10.1007/s00464-022-09692-y.

Gastric outlet obstruction (GOO) is a condition secondary to mechanical obstruction and is characterised by post-prandial abdominal pain, abdominal distension, nausea and vomiting. These symptoms usually result in nutritional compromise and detriment to a patient’s quality of life (QOL). GOO can be caused by both benign and malignant pathologies, with a preponderance for malignancies [1, 2]. If primary surgery to remove the underlying aetiology is not possible, endoscopic enteral stenting (ES) or surgical gastrojejunostomy (S-GJ), either via an open or laparoscopic approach, are established methods of treating GOO [3, 4].

The decision to pursue one option over the other should take into account factors such as the patient’s life expectancy, fitness for surgery, anticipated duration of post-procedural recovery and relative likelihood of success of either modality. Meta-analyses have demonstrated both ES and S-GJ to be clinically effective in treating GOO but ES is associated with increased re-intervention rates, largely due to tumour ingrowth through the stent thus compromising its patency. Currently, S-GJ is considered in patients with good performance status and life expectancy greater than 3 months although this decision has to factor in the recovery process, post-operative morbidity and potential adverse events [5–7].

Endoscopic ultrasound guided gastrojejunostomy (EUS-GJ) with a lumen apposing metal stent (LAMS) has recently emerged as an alternative to ES and S-GJ in the management of GOO. EUS-GJ combines the minimally invasive nature of ES with the creation of a conduit between the stomach and the jejunum away from the primary tumour site, in similar fashion to a S-GJ, thus preventing compromise of the stent patency by tumour ingrowth, if performed in patients with malignant GOO [8]. Meta-analyses have demonstrated EUS-GJ to be an efficacious and safe modality in treatment of GOO. [9, 10] In this study, we aimed to analyse the efficacy, safety and long-term outcomes of all patients who underwent EUS-GJ at our institutions.

## Methods

### Study design

We conducted a multi-centre, retrospective review of all consecutive patients who underwent EUS-GJ between August 2018 and March 2021 across three tertiary institutions in the United Kingdom: Leeds Teaching Hospitals NHS Trust (LTHT), University Hospital Southampton NHS Foundation Trust and University College London Hospitals NHS Foundation Trust. Data on demographics, baseline patient characteristics, procedural information and post-procedural events were obtained from each institution’s electronic records database. The study was registered as a service evaluation project and approved by the information governance board at LTHT and was carried out in accordance with the Helsinki Declaration.

### Patient selection

All patients were deemed not suitable for S-GJ on the basis of poor fitness for surgery and/or surgical technical factors such as an unfavourable anatomy. Suitability for EUS-GJ was made following discussion in a formal multi-disciplinary team setting or close liaison between oncology, surgical and endoscopic teams.

### Lumen apposing metal stents (LAMS)

LAMS are fully covered stents, made of nitinol material, which are configured in a dumbbell shaped design with two flanges on either end providing lumen to lumen apposition when fully deployed. This unique design allows the LAMS to safely function as a conduit as the apposition capability of the flanges provide traction between two lumens to bring them together and the fully covered component of the stent prevents any leakage within the stent or around the tract. The addition of an electrocautery enhanced delivery system through which the LAMS can be deployed has simplified what used to be a multi-step procedure requiring exchanges of different endoscopic accessories. The first mainstream role of LAMS was in endoscopic drainage of pancreatic fluid collections (PFC) but LAMS are increasingly used in EUS guided biliary drainage (EUS-BD) and the creation of internal digestive anastomoses, particularly EUS-GJ [11]. The Hot AXIOS™ [Boston Scientific, Massachusetts, USA] LAMS was used in our study.

### EUS-GJ procedure

The procedures were performed by five senior interventional endoscopists with prior extensive experience with EUS and the use of LAMS in the management of PFC and EUS-BD. All procedures were performed with the patient under general anaesthesia with endotracheal intubation. Prophylactic antibiotics were given in accordance with individual institution’s policy. In this study, the EUS-GJ procedure was carried out via the following method:

1. Identification of a jejunal loop under EUS guidance. In some instances, this was assisted by endoscopic insertion of an oroduodenal/orojejunal catheter downstream of the level of obstruction and infusion of a mixture of contrast, distilled water and methylene blue dye via an irrigation pump, thus distending the jejunum and improving visibility on EUS and x-ray screening. On occasion, the endoscopist may choose to confirm the presence of a jejunal loop (rather than colon) by transgastric puncture using a 22G fine needle aspiration (FNA) needle and aspiration of methylene blue stained jejunal contents.
2. Deployment of the LAMS to create a gastrojejunostomy was performed either via the freehand or wire-guided approach:
  (i) Freehand approach: Direct puncture through the gastric wall and into the jejunum with the cautery enhanced tip of the LAMS delivery system
  (ii) Wire-guided approach: Puncture of the jejunum with a 19G FNA needle, passage of a guidewire through the needle into the jejunum and loading the LAMS delivery system over the guidewire and puncture through the gastric wall and into the jejunum with the cautery enhanced tip of the LAMS delivery system
3. Following deployment of the flange of the LAMS on the gastric side, an endoscopic view of methylene blue stained solution refluxing back into the stomach through the lumen of the LAMS and/or injection of contrast through the LAMS from the stomach into the jejunum confirms successful creation of a gastrojejunostomy.
4. At one institution (LTHT), endoscopic balloon dilatation through the lumen of the LAMS was routinely performed with a balloon diameter of 18 mm–20 mm in increments of 1 mm. This practice was abandoned following displacement of the LAMS during balloon dilatation.

### Definitions

Technical success was defined as the successful creation of a gastrojejunostomy with the LAMS. Clinical success was defined as an improvement of oral enteral intake and increased tolerance of a higher consistency diet when compared to baseline, utilising a validated scale of dietary consistencies (no diet = 0, liquids = 1, semi-solids = 2, low-residue diet/solids = 3), known as the gastric outlet obstruction scoring system (GOOSS) [12]. Patients who did not have a technically successful procedure were not included in the analysis of clinical success rates. Adverse events were defined in accordance with the American Society of Gastrointestinal Endoscopy lexicon for reporting endoscopic adverse events [13].

### Statistics

Mean values are expressed with standard deviation values and median values with range values. Comparison of GOOSS pre and post-procedure was performed with the Wilcoxon signed-rank test. Statistical significance was determined at a *p*-value of less than 0.05. Statistical analyses were performed with IBM SPSS Statistics version 26.0 (IBM Corp., Armonk, NY, USA).

## Results

Twenty five patients (15 males) with a median age of 63 (range 29–80) years old were included for analysis. Demographics and baseline characteristics of the patients are summarised in Table 1. The aetiology of GOO was malignancy in 22 patients, with 10 patients having underlying pancreatic cancer. Oral intake was compromised in all 25 patients. Nine patients could not tolerate any intake, 15 patients could only tolerate liquids and 1 patient could tolerate semi-solids. Supplementary feeding was required in 11 patients prior to the intervention (nasojejunal tube feeding = 1, total parenteral nutrition = 10).

**Table 1:** Demographics and baseline characteristics of the patients

Characteristic	Value
Mean age (SD)	61.4 (14.3)
Gender	15 M: 10F
Mean body mass index (SD)	23.1 (4.49)
Aetiology of GOO	
Malignant	88% (22/25)
Pancreatic cancer	40% (10/25)
Duodenal/ampullary cancer	12% (3/25)
Gastric cancer	4% (1/25)
Cholangiocarcinoma	4% (1/25)
Metastases from other primaries	28% (7/25)
Benign	12% (3/25)
Chronic pancreatitis	8% (2/25)
Peptic stricture	4% (1/25)
Post-surgical anatomy	
Prior Whipple’s and liver transplant	4% (1/25)
Location of stricture	
Pre-pyloric/pyloric	8% (2/25)
First/second duodenal portion	60% (15/25)
Third/fourth duodenal portion	28% (7/25)
Proximal jejunum	4% (1/25)
Prior duodenal stenting	20% (5/25)
Reason not for S-GJ	
Deemed unfit for surgery	84% (21/25)
High surgical risk due to disease or vascular factors	12% (3/25)
Post-surgical anatomy	4% (1/25)
Baseline GOOSS	
0	36% (9/25)
1	60% (15/25)
2	4% (1/25)
3	0%
Baseline mean GOOSS (SD)	0.68 (0.57)
Supplementary feeding	
Total parenteral nutrition	40% (10/25)
Nasojejunal tube feeding	4% (1/25)

### Key outcomes

Key outcomes are summarised in Table 2. Technical success was achieved in 23 of 25 patients. Initial stent misplacement occurred in two patients, both of whom underwent the freehand technique. In the first patient, the distal flange of the LAMS was opened in the peritoneum which was recognised immediately by visualisation of the peritoneal lining through the lumen of the LAMS. The LAMS was removed and endoscopic closure of the gastric defect with performed with clips. A second LAMS was placed successfully; therefore leading to technical success in creation of a gastrojejunostomy. In the second patient, stent placement was attempted twice. During both attempts, the distal flange was deployed outside the wall of the jejunum. This may be due to the mobility of the jejunum which was displaced with compromised endosonographic views during attempted puncture with the stent delivery system. Both gastric defects were closed with endoscopic clips. The procedure was abandoned in this instance, although the patient successfully underwent a EUS-GJ at a later date which was performed by a different operator. In both cases, prophylactic broad spectrum antibiotics were commenced and the patients did not develop symptoms of peritonitis.

**Table 2:** Key clinical and procedural outcomes

Characteristic	Value
Technical success *n* (%)	92% (23/25)
EUS-GJ technique used *n* (%)	
Freehand approach	68% (17/25)
Freehand approach and orojejunal irrigation	8% (2/25)
Wire-guided approach	4% (1/25)
Wireguided approach and orojejunal irrigation	20% (5/25)
Clinical success *n* (%)	100% (23/23)
Diameter of LAMS *n* (%)	
15 mm	16% (4/25)
20 mm	84% (21/25)
Balloon dilatation of lumen of LAMS *n* (%)	20% (5/25)
Oral intake at 30 days post-procedure or first follow-up	
0	0
1	8.7% (2/23)
2	34.8% (8/23)
3	56.5% (13/23)
Post-procedural mean GOOSS (SD)	2.52 (0.59)
Adverse event rate *n* (%)	8% (2/25)
30-day mortality rate *n* (%)	8% (2/25)
Re-intervention rate *n* (%)	4.8% (1/21)

In a third patient, a successfully placed LAMS was displaced following endoscopic balloon dilatation through the lumen of the LAMS. The gastric defect was closed with endoscopic clips and duodenal stent placed instead.

Clinical success was achieved in all 23 patients who had a technically successful procedure. All patients who had required supplementary feeding were successfully weaned off them. The median time from LAMS deployment to cessation of supplementary feeding was 5 days (IQR 4–7 days). There was a significant improvement of mean GOOSS (2.52 ± 0.59 vs 0.68 ± 0.57, *p* < 0.01).

Adverse events occurred in 2 patients; both of which were classed as moderate due to an extended hospitalisation of 5 days for prophylactic antibiotics to prevent peritonitis in two patients who had stent misplacements requiring endoscopic closure of the gastric defect. Two patients died within 30 days of the procedure, but mortality was unrelated to the EUS-GJ procedure itself. The first patient had a technically successful procedure but developed obstructive uropathy from the underlying tumour process leading to renal failure. The patient declined nephrostomies and died 5 days after the EUS-GJ procedure. The second patient also had a technically successful procedure but died 28 days following due to progression of the underlying malignant disease.

Long-term follow-up data were available for 21 of 23 patients who had a technically successful EUS-GJ over a median follow-up period of 162 days (IQR 63–286). Only one patient required re-intervention. This was in a patient who developed recurrent GOO symptoms and underwent endoscopic re-intervention 63 days after the initial EUS-GJ. At the time of repeat endoscopy, there was preferential passage of the endoscope and contrast through the lumen of the LAMS into the afferent limb of the jejunum due to the angle of the LAMS. This was successfully rectified by placement of a long colonic stent into the direction of the efferent limb and fixated to the LAMS with endoscopic clips. The patient resumed oral intake and no further re-intervention was required.

## Discussion

Traditionally, the two main modalities for treatment of GOO were endoscopic ES or a S-GJ via either an open or laparoscopic approach. The recent innovations in therapeutic endoscopy continue to push the boundaries of the limits of minimally invasive options for patients. The potential of EUS in the internal creation of digestive anastomoses and the advent of new technology, such as the LAMS in this case, have led to the possibility of EUS-GJ, which is now being increasingly recognised as a viable option in the treatment of GOO.

In this multi-centre, retrospective series, we demonstrated EUS-GJ to be an effective and safe treatment option for patients with GOO. Prior to the intervention, all of the patients exhibited significant nutritional compromise, consequentially rendering them to be suboptimal physiologically for a S-GJ. Prior to consideration for an EUS-GJ, a multi-disciplinary approach involving the endoscopist, surgeon, nutritionist and oncologist (if applicable) is essential to determine the most appropriate modality of treatment for a patient with GOO.

One important advantage of EUS-GJ over its counterparts is its applicability in patients with altered post-surgical anatomy of the alimentary tract. Perez-Miranda et al., reported the successful completion of EUS-GJ in seven patients with previous surgeries (Whipple’s = 5, roux-en-y = 1, partial gastrectomy = 1) as part of a larger retrospective comparison of EUS-GJ and S-GJ [14]. EUS-GJ has also been reported to be an efficacious treatment for both benign and malignant causes of afferent loop syndrome in patients with post-surgical anatomies [15, 16]. In the present series, EUS-GJ was performed successfully in one patient who, 5 years previously, had undergone a liver transplant and subsequent Whipple’s procedure after discovery of a cholangiocarcinoma but developed GOO secondary to extrinsic compression from recurrence of the malignancy.

There are several reports in the literature evaluating EUS-GJ against ES and S-GJ. Chandan, et al. conducted a meta-analysis of 659 patients in five retrospective studies comparing outcomes of EUS-GJ and ES. They demonstrated comparable technical success (EUS-GJ 95.2% vs ES 96.9%), clinical success (EUS-GJ 93.3% vs ES 85.6%), although ES was more likely to be associated with higher re-intervention rates (EUS-GJ 4% vs ES 23.6%) [17]. However, three of the five studies were published as abstracts without availability of further data. Furthermore, the specific EUS-GJ technique employed was variable across the included studies and not described in some. The authors acknowledged these limitations and given the relative novelty of EUS-GJ, reflects the paucity of dedicated EUS-GJ studies and the variation in reporting of techniques and outcomes.

Bronswijk, et al. conducted a propensity score-matched comparator, multi-centre, retrospective study between EUS-GJ and laparoscopic S-GJ demonstrating similar technical and clinical success rates, although patients who underwent EUS-GJ resumed oral intake quicker, had a shorter length of stay and encountered less adverse events [18]. Perez-Miranda, et al. conducted a retrospective, international, multi-centre analysis comparing outcomes and cost modelling between 29 patients who underwent laparoscopic S-GJ and 25 patients who underwent EUS-GJ. Similar technical success (S-GJ vs EUS-GJ: 100% vs 88%; *p* = 0.11) and clinical success (S-GJ vs EUS-GJ 90% vs 84%; *p* = 0.11) were observed in both groups. However, EUS-GJ may be more cost beneficial with the financial analysis demonstrating EUS-GJ to be a third the cost of a laparoscopic S-GJ.

Although EUS-GJ is increasingly adopted, its place in the algorithm of management of GOO alongside the more established ES and S-GJ modalities remains unclear. ES remains an effective, safe procedure which can be performed in most secondary care institutions. The need for re-intervention following ES tends to be predicted by an anticipated longer life expectancy of the patient. Although re-intervention is usually straight forward, the need for repeated procedures, symptom recurrence affecting QOL and interruptions in oncological treatment have to be considered. A life expectancy threshold of 3 months has been suggested as a cut-off for considering EUS-GJ or S-GJ over ES [19]. In the present study, 22 patients had malignant GOO and over a median follow-up period of 161 days, re-intervention was only required in 1 patient. Eight patients were still alive at the end of the follow-up period. This reflects the patient selection process which identifies patients suitable for an EUS-GJ instead of ES on the basis of a reasonable life expectancy, even in the setting of malignant GOO. Prospective, randomised controlled trials evaluating EUS-GJ and ES for treatment of malignant GOO are underway and the results will be eagerly awaited [8, 20]. Until then, a personalised approach should be adopted, accounting for individual patient and disease characteristics, as well as the availability of local resources and technical expertise.

The long-term impact and any potential safety issues of a LAMS remaining in-situ remains unclear. In this series, no adverse events relating to a successfully placed LAMS were encountered over the follow-up period (range 5–474 days). Delayed or late safety events following placement of LAMS for drainage of pancreatic fluid collections have been well documented, particularly with regards to bleeding [21]. In the context of pancreatic fluid collections, bleeding occurs due to erosion of the LAMS through the wall of the decompressed cavity or any surrounding vasculature. Irani et al. described occurrences of stent induced ulcerations and granulation tissue hypertrophy causing structuring in their series of LAMS used to treat benign gastrointestinal tract strictures [22]. Taibi et al. reported a case of delayed perforation of a jejunal loop adjacent to the LAMS 6 months after an EUS-GJ was performed in a patient with cystic duodenal dystrophy [23]. Therefore, the concern of hypothetical long-term safety issues of LAMS has to be contextualised accordingly to the nature of GOO and site of LAMS placement. Nevertheless, the safety of LAMS in EUS-GJ should be evaluated with prospective, long-term follow-up data.

EUS-GJ remains an advanced endoscopic technique which is usually only performed in tertiary institutions. There is a substantial learning curve even for endoscopists with extensive experience in diagnostic and therapeutic endoscopy [24]. In the current climate of centralisation of tertiary services, EUS-GJ is likely to only be performed by a select group of expert endoscopists. The importance of a learning curve in the application of novel endoscopic techniques is reflected in a multi-centre, retrospective study of the outcomes of EUS-GJ by Kastelijn, et al. [25] They demonstrated an adverse event rate of 26.7% (12/45), of which 5 (11.1%) resulted in fatalities. All fatal events occurred in one centre and early in the study period, likely reflecting individual learning curves and also refinement of the technique of EUS-GJ over time. Due to the retrospective nature of our study, it was not designed to evaluate individual operator’s learning curve. However, all operators have an extensive experience of use of LAMS in other indications such as drainage of pancreatic fluid collections, therefore, possessing such experience as a baseline before performing EUS-GJ would be advisable.

There are multiple varieties of the technique of performing a EUS-GJ [8] and a dedicated balloon device to anchor the jejunal loop for puncture has also been developed, although is not widely available yet [26]. It is conceivable that techniques and complementary devices for EUS-GJ will continue to be refined with the passage of time. Furthermore, there has been cumulative international experience of stent misdeployments and the employment of different strategies to rectify them. Ghandour et al. reported on the classifications, outcomes and managements of misdeployed stents in 467 patients across 16 institutions [27].

There are several limitations to our study. Firstly, this was a retrospective series and therefore subject to inherent pitfalls of it’s design. Secondly, as patients were identified from multiple institutions without pre-defined enrolment criteria, there would be differences in patient selection for the procedure. Thirdly, the lack of a control group precluded direct comparison of outcomes although all consecutive patients were included for analysis, which may mitigate a degree of selection bias. Lastly, all procedures were performed by experienced endoscopists at tertiary institutions therefore the results may not be generalisable.

In conclusion, EUS-GJ appears to be an effective and safe procedure when performed by experienced endoscopists. Prospective studies in the form of randomised, controlled trials are needed to evaluate the performance of EUS-GJ against its counterparts.

## Supplementary Information

Below is the link to the electronic supplementary material.

Supplementary file1 (MP4 184116 KB) Video 1 depicts key components of EUS-GJ
